# Barriers and enablers to the provision and receipt of preoperative pelvic floor muscle training for men having radical prostatectomy: a qualitative study

**DOI:** 10.1186/1472-6963-13-305

**Published:** 2013-08-13

**Authors:** Andrew D Hirschhorn, Gregory S Kolt, Andrew J Brooks

**Affiliations:** 1 School of Science and Health, University of Western Sydney, Sydney, Australia; 2 Westmead Private Physiotherapy Services, Sydney, Australia; 3 Western Urology, Sydney, Australia

**Keywords:** Prostatectomy, Urinary incontinence, Translational research, Qualitative research, Physical therapy modalities

## Abstract

**Background:**

Strong evidence exists to support preoperative pelvic floor muscle training (PFMT) to reduce the severity and duration of urinary incontinence after radical prostatectomy. Receipt of preoperative PFMT amongst men having radical prostatectomy in Western Sydney, however, is suboptimal. This study was undertaken to investigate barriers and enablers to provision/receipt of preoperative PFMT from the perspectives of potential referrers to and providers of PFMT, and of men having radical prostatectomy.

**Methods:**

A qualitative research design was used. Semi-structured, one-to-one interviews were conducted with participants from three groups: (i) current and potential referrers to PFMT, including urological cancer surgeons, urological cancer nurses and general practitioners (n = 11); (ii) current and potential providers of PFMT across public and private sector hospital and outpatient settings, including physiotherapists and continence nurses (n = 14); and (iii) men having had radical prostatectomy at a specific public and co-located private hospital in Western Sydney (n = 13). Interview schedules were developed using Michie’s theoretical domains for investigating the implementation of evidence-based practice, and allowed participants to identify potential and actual barriers and enablers to preoperative PFMT. Transcribed interview data were analysed using a framework approach, and key themes were identified.

**Results:**

Participant groups concurred that a recommendation for PFMT from the urological cancer surgeon, accompanied with a referral to a specific provider, was a key enabler of preoperative PFMT. Perceived barriers varied between participant groups and across public and private healthcare settings. Perceptions of financial cost of private sector PFMT, limited knowledge amongst referrers of public sector providers of PFMT, and limited awareness amongst patients of the benefits of PFMT were all posited to contribute to suboptimal PFMT provision and receipt.

**Conclusions:**

This study has provided valuable data on barriers and enablers to preoperative PFMT, with implications for the planning of a behaviour change intervention to improve provision and receipt of preoperative PFMT in Western Sydney.

## Background

Prostate cancer is the most common form of malignancy in Australian men, with more than 20,000 new cases diagnosed in 2008 [1]. In approximately 90% of new prostate cancer diagnoses, the cancer is localised or ‘locally advanced’ (i.e. the cancer is confined to the prostate gland or prostate region) [2]. Conventional treatments for localised prostate cancer include active surveillance, radical prostatectomy and radiotherapy (external beam and seed brachytherapy) [3]. While guidelines for choice of treatment are not ‘clear-cut’, radical prostatectomy may be preferentially indicated for patients with a greater life expectancy and who are fit for surgery [4]. The majority of men aged less than 70 to 75 years and diagnosed with localised prostate cancer, certainly in Australia and the USA, have radical prostatectomy as primary treatment [3,5]; more than 6,000 radical prostatectomies are performed in Australia annually [6].

Advantages of radical prostatectomy over active surveillance and radiotherapy include improved long-term cancer control and ability to determine prognosis according to pathologic cancer features [3]. Urinary incontinence, however, is a common complication of radical prostatectomy. The reported incidence of post-prostatectomy urinary incontinence (PPUI) varies according to clinical definition and time of follow-up; recently published case-series report PPUI rates of 0 to 30% at twelve months after surgery (defined as using > 1 continence pad/day) [7], but up to 87% of patients report PPUI symptoms and/or bother at three months [8]. PPUI, whether transient or persistent beyond twelve months, reduces health-related quality of life, and may delay return to work and/or normal physical and social activity [9].

While the precise aetiology of PPUI may vary between patients, urodynamic studies demonstrate that bladder/urethral sphincteric incompetence, resulting from surgical trauma, is a contributing factor in > 90% of cases [7]. Prolapse of the urethra through the pelvic floor may further impair residual sphincter function after radical prostatectomy [10]. Contraction of the pelvic floor muscles, specifically the rhabdosphincter-levator ani complex, moves to close and elevate the urethra, potentially compensating for this sphincter incompetence and dysfunction in periods of urinary ‘stress’. Hence pelvic floor muscle training (PFMT), whereby patients are taught by healthcare providers to voluntarily contract the pelvic floor muscles, with or without biofeedback, is a well-described conservative treatment for PPUI. While there is equivocal evidence of benefit for PFMT commenced *after* radical prostatectomy [8,11], there is increasing Level 1 evidence to support PFMT, commenced *preoperatively*, to reduce the severity and duration of PPUI [12-16]. Consequently, published recommendations for the conservative management of PPUI include that all men having radical prostatectomy receive preoperative PFMT, preferably from a physiotherapist or continence nurse [17].

Provision/receipt of preoperative PFMT for men having radical prostatectomy in Australia has not previously been reported. Preliminary audit data collected by the authors demonstrates, however, that in our clinical setting (i.e. a tertiary referral urological cancer centre in Western Sydney, Australia) approximately 60% of men having radical prostatectomy do not receive preoperative PFMT. Given significant urban–rural differences in access to other prostate cancer-related services in Australia [18], receipt of preoperative PFMT is likely to be even lower in settings outside major Australian cities.

French et al. have described a systematic, four-step approach to developing theory-informed behaviour change interventions to implement evidence into clinical practice [19], e.g. to improve provision/receipt of preoperative PFMT. The first two steps of this approach are: (i) to identify the problem, i.e. identify ‘who needs to do what differently’; and (ii) to assess the problem, i.e. identify those local barriers and enablers that need to be addressed. In the present study, we investigated local barriers and enablers to preoperative PFMT amongst potential referrers (e.g. urological cancer surgeons), providers (e.g. physiotherapists) and patients (men having radical prostatectomy) in both public and private healthcare sectors. These findings were to be used to inform a multifaceted behaviour change intervention aimed at increasing the rate of provision/receipt of preoperative PFMT in our clinical setting.

## Methods

### Study design

A qualitative analysis of semi-structured, one-to-one interviews with a purposive sample of: (i) current and/or potential referrers to preoperative PFMT, henceforth ‘referrers’; (ii) current and/or potential providers of preoperative PFMT, henceforth ‘providers’; and (iii) men having undergone radical prostatectomy, henceforth ‘patients’.

### Setting

The study was designed to explore barriers and enablers to provision of preoperative PFMT for men having radical prostatectomy in a specific health service, consisting of one tertiary referral public hospital and one geographically co-located private hospital in Western Sydney, one of the largest population areas of Australia. The study was undertaken as a component of a broader project aimed at improving the implementation of preoperative PFMT into clinical practice. Interviews took place between August 2011 and February 2012. Ethical approval for the study was obtained from Western Sydney Local Health District’s Human Research Ethics Committee (HREC2011/5/4.7(3311) AU RED HREC/11/WMEAD/87) and University of Western Sydney’s Human Research Ethics Committee (H9195).

### Participants and recruitment

Participants were drawn from three defined groups:

(i) Referrers: consisting of urological cancer surgeons (public and private sector), urological cancer nurses (public and private sector) and general practitioners (GPs).

Key local informants, including all urological cancer surgeons, were identified by the researchers, and provided with individual letters of invitation to participate. Open invitations to participate were also made at Department of Urology clinical service meetings (public hospital) and at a GP continuing education session focused on urology and prostate cancer (private hospital).

(ii) Providers: consisting of physiotherapists (public and private sector, inpatient and outpatient settings), continence nurses (public sector outpatient setting), and their managers.

Key local informants (e.g. senior physiotherapists in continence, urology and outpatient care settings) were again identified by the researchers and provided with individual letters of invitation to participate. Recruitment posters were also placed in local community health centres known to provide a continence services, and the public and private hospital urology wards and physiotherapy departments.

(iii) Patients: consisting of men undergoing radical prostatectomy.

All men undergoing radical prostatectomy at both the public and private hospitals over a three-month period were approached while in hospital (by third parties to the research), to provide consent to receive an invitation to participate. Invitations were posted three months postoperatively. An open invitation to participate was also made at a meeting of the local Prostate Cancer Support Group.

Where participants identified additional key informants (e.g. additional providers of PFMT), these informants were subsequently invited to participate.

### Interviewer background and data collection

All interviews were conducted by a single researcher (ADH). External to his research role, ADH maintains a combined clinical/managerial appointment within a private-sector physiotherapy group in Western Sydney that provides physiotherapy services to men having radical prostatectomy in both inpatient and outpatient settings. As such, many of the referrers and providers interviewed for the study knew or had come into contact with ADH through the course of their clinical practice.

Where possible, interviews were conducted in a research office adjacent to the private hospital. Where participants were unable to attend the research office (e.g. for reasons of work), interviews were conducted within their work offices, or in the case of patients, their homes. Two patients living in regional areas were interviewed by telephone. Interviews were audio-recorded for subsequent transcription. Participants provided written informed consent for recording of interviews and use of anonymised quotations.

### Interview schedules

Interview schedules were developed primarily to elicit responses about factors that might act as barriers and enablers to PFMT. Separate interview schedules were developed *a priori* for each participant group, however were flexible, and evolved as the study progressed. Because of varying definitions of PFMT in the literature, all participants were provided with an outline of published recommendations on PFMT for men having radical prostatectomy, before being interviewed [17]. This outline defined three key aspects of PFMT from the perspective of the researchers (i.e. that it be commenced preoperatively, that it be supervised by a physiotherapist or continence nurse, and that it include observation-based feedback).

(i) Referrers

Referrers were asked to describe their current healthcare position, their experience, and any specialist qualifications held in the management of men with prostate cancer and/or with urinary incontinence. In order to map the process that men follow in their healthcare before and after radical prostatectomy, referrers were also asked to describe their specific role in the care of men undergoing radical prostatectomy, and how men are referred to their care. Further, referrers were asked to describe their current practices as regards referral of men having radical prostatectomy for PFMT.

Michie’s twelve theoretical domains, component constructs, and eliciting questions for investigating the implementation of evidence-based practice were used to guide questioning regarding barriers and enablers to PFMT for referrers (and providers) [20]. These theoretical domains (e.g. ‘knowledge’, ‘skills’, and ‘social/professional role and identity’) were initially derived through a consensus process involving health psychology theorists, health services researchers and health psychologists, the aim of the process being to develop a framework of use to researchers/health services managers seeking to implement evidence-based practices (e.g. preoperative PFMT) [20]. Noting that ‘referrers’ in this study by definition do not provide preoperative PFMT, the specific questions asked related to both: (i) barriers and enablers to their *referral* of men for PFMT, as well as (ii) their perceptions as regards subsequent barriers and enablers to PFMT *provision/receipt*.

(ii) Providers

The interview schedule for providers followed a similar format to that described for referrers. As it was acknowledged that the ability of providers to indeed *provide* PFMT was also dependent on referrers and the patients themselves, providers were also given an opportunity to discuss their perceptions of barriers and enablers to PFMT from the perspectives of these other participant groups.

(iii) Patients

The interview schedule for patients consisted of five component topics: demographics, including details of their surgery (i.e. surgeon, hospital, date); their home, transport and work arrangements; their receipt or otherwise of PFMT; their decision-making (as applicable) around PFMT; and their perceptions of the barriers and enablers to PFMT. No specific theory was used to guide the interview schedule for patients. Again, in order to map the process men follow in their healthcare before and after surgery, patients were also asked to describe their path from initial testing to diagnosis of localised prostate cancer, radical prostatectomy, and perioperative care.

### Analysis

Audio-recordings of interviews were saved as password-protected computer files, and transcribed verbatim using transcription software (DSS Player Version 7 Plus, Olympus Imaging Corporation). Transcribed interview data were analysed using a framework approach [21], consisting of the following five stages: (i) *familiarisation* with the raw data to list key ideas and recurrent themes; (ii) *identifying a thematic framework;* (iii) *indexing*, whereby the thematic framework is applied systematically to the raw data; (iv) *charting*, whereby data is rearranged according to the relevant thematic domain to form charts; and (v) *mapping and interpretation*. Specifically, printed and computer files of transcribed interviews were reviewed on two separate occasions to generate a preliminary list of themes (e.g. for the participant group: ‘lack of knowledge’ and ‘availability of services’). Noting that Michie’s theoretical domains were used to guide interview schedules, it was clearly identified that the generated themes correlated strongly with said domains (e.g. ‘knowledge’ and ‘environmental constraints’). As such, relevant participant responses/statements were again reviewed, thence indexed by theoretical domain, and copied and pasted into Excel spreadsheets/charts of domains, stratified by participant group and whether the statement related to a perceived barrier, enabler, or both. Finally, charts were reviewed, and responses/statements were compared between and across theoretical domains and participant groups. Transcription and analysis were undertaken by ADH. Analysis, and the derivation of themes, was discussed between all researchers for agreement.

## Results

Overall, 38 participants were interviewed, 11 referrers, 14 providers and 13 patients. Participant characteristics are summarised in Table 1. All participants were interviewed once only. Interviews were of mean 36 ± 8 minutes duration (range 19 to 52 minutes).

**Table 1:** Participant characteristics

**Participant group**		**Total**	**Private sector**	**Public sector**
**Referrers**	Urological cancer surgeons	3	3^a^	3^a^
	Urological cancer nurses	5	3	2
	General practitioners	3	2^b^	3
**Providers**	Physiotherapists (overall)	10	5	5
	Inpatient setting (urology and/or continence)		4^c^	3^c^
	Outpatient setting (general and/or continence)		4^c^	4^c^
	Continence nurses	4	0	4
**Patients**		13	10	3

^a^All urological cancer surgeons maintained private and public hospital appointments.

^b^Two general practitioners maintained a private hospital appointment.

^c^Three private sector physiotherapists and one public sector physiotherapist maintained both inpatient and outpatient roles.

The factors that participants identified as barriers and enablers to preoperative PFMT are presented below. Factors are categorised by theoretical domain, and where appropriate, the perceptions of each participant group are presented separately. Sample quotations are presented in Table 2 (barriers) and Table 3 (enablers).

**Table 2:** Quotations describing barriers to provision/receipt of preoperative PFMT

**1. Social/Professional role&beliefs about capabilities**	
*(There is) a very limited role (for GPs to influence provision/receipt of PFMT), because again, it’s got to be initiated by the specialists.*	*(General Practitioner 2)*
*The pathway today, we find that it's a little bit difficult is, once the patient get's onto (a) specialist's care, we find often we don't see the patient coming back to us for a little while, and quite often it's… already (having had) a prostatectomy.*	*(General Practioner 3)*
*I just don't get the referrals to see (men for PFMT), which is probably because doctors are not aware that I provide the treatment, which is again, probably because I never publicise that I provide it.*	*(Physiotherapist (Public Sector) 1)*
*… there I suppose hasn't been an opportunity to treat male patients. I think it's through tradition that we have just received female referrals, and hence there hasn't been any consultation with specialists, nurses,etc*. *who are involved in patients who have prostatectomies…*	*(Physiotherapist (Public Sector) 3)*
*There's no reason that people … assuming they've been given the correct information, cannot get the appropriate exercises either through the DVDs, which are available off the net and through the Cancer Association or Council or whatever it is, and take it from there.*	*(Patient 3)*
**2. Knowledge/skills**	
*And there’s no facility available in the public sector, for them to participate in preoperative pelvic floor exercises.*	*(Urological Cancer Surgeon 3)*
*I don't think they (GPs) appreciate the situation (that urinary incontinence may be a complication of radical prostatectomy). They only know what they read in the lay media.*	*(General Practitioner 2)*
*There are no formal qualifications in men's health that you can get, like there are for women's health, so for example, there is a postgraduate program for women's health and continence in Melbourne, there's no male equivalent for that…*	*(Physiotherapist (Private Sector) 5)*
*They (the surgeon) just said, 'you have prostate cancer, what are you going to do about it?' And then you had to make a choice out of the options they gave you, but nothing was ever said about pelvic floor exercises.*	*(Patient 2)*
*I've heard of pelvic floor exercises before, but they're all for women. They're not things that men do.*	*(Patient 1)*
**3. Environmental context and resources**	
*It's just staffing, it's our lack of resources that make it difficult… which would make it difficult with a new service. Not difficult, (but) challenging. Staff.*	*(Physiotherapist (Public Sector) 4)*
*I mean some of these public patients are really poor, they don't have any money. And you can't get blood out of a stone, so if they haven't got the money, they don't do it.*	*(Urological Cancer Surgeon 3)*
*Some of these guys are so obsessed with their daily timetables they barely have enough time to take time off to do their operation, let alone do other things that they don't perceive are as important, perhaps as important as they are.*	*(Urological Cancer Surgeon 3)*
**4. Memory, attention and decision processes**	
*… a diagnosis of prostate cancer is confronting for most men, and then the discussion on the treatment and how it affects the quality of life is very daunting. And in my experience, the obsession with cancer and the obsession with getting rid of the cancer tends to dominate the whole focus.*	*(Urological Cancer Surgeon 3)*
*And that’s where I think a lot of it falls through, that you’re missing it (the referral to a provider of PFMT) at the front, where the (receptionist are), they’re so busy…*	*(Urological Cancer Nurse 1)*
**5. Social influences (Norms)**	
*Look, I haven't been instructed to see men. So I haven't been asked by… we haven't had any pressure put on from elsewhere, onto my boss, who hasn't then put pressure onto me.*	*(Physiotherapist (Public Sector) 2)*
**6. Beliefs about consequences**	
*… if you take on a new service, then what happens to your other acute patients? So then you need to either increase your staffing, or increase your KPI (key performance indicator) for your other acute patients, so you’re dropping the standard.*	*(Physiotherapist (Public Sector) 3)*
**7. Additional patient-related barriers**	
*There are patients who … just want to do the bare minimum. There are patients to whom you even say, ‘Look, you’ve got cancer, you need to be operated on, …,’ they don’t care.*	*(Urological Cancer Surgeon 2)*

**Table 3:** Quotations describing enablers to provision/receipt of preoperative PFMT

**1. Social/Professional role&beliefs about capabilities**	
*(Regarding referring patients for PFMT) (it) routinely occurs for every patient, private and public, preoperatively. As soon as a decision is made for surgery, in some cases even before the decision for surgery has been made.*	*(Urological Cancer Surgeon 1)*
*What we've found dealing with a few urologists, the ones that do refer the surgeons straight through will present to treatment. … I'm assuming here, but I think that anything that their urologist will tell them, they will do.*	*(Physiotherapist (Private Sector) 2)*
*I suppose for our practice, it's a specific service that we offer. And I suppose the resources and the amount of work that's gone into developing the educational packages makes it, I suppose, a lot easier for us to provide the service.*	*(Physiotherapist (Private Sector) 4)*
*… there is a reliance on us for certain surgeons to really focus on this (PFMT) and get it absolutely right, and also to make sure patients have had feedback about their knowledge of performance, about their ability to do these exercises specifically, and how well they're doing them.*	*(Physiotherapist (Private Sector) 5)*
**2. Knowledge/skills**	
*There's a good paper that is currently being reviewed … that shows that preoperative pelvic floor exercises make a significant difference to postoperative time of incontinence and also grade of incontinence. There's also a randomised study that shows that preoperative pelvic floor exercises make a difference to postoperative incontinence. And so those two studies are probably the main reasons why I do it (refer to PFMT).*	*(Urological Cancer Surgeon 1)*
*… so just hearing men's stories, that come back month after month and year after year, and reporting back on where they were and where they are, there certainly is evidence that… that pelvic floor exercises really do work.*	*(Urological Cancer Nurse 2)*
*Unless he (the surgeon) had said anything about it (PFMT), I wouldn't have even dreamt about it, having it. So what I would say to you, is that it comes back to your specialist.*	*(Patient 8)*
**3. Environmental context and resources**	
*… most of the patients that I've seen are reasonably close by, because the referrals we receive are from the urologists working out of (the same suburb).*	*(Physiotherapist (Private Sector) 3)*
*I think it's relatively easy (to provide PFMT), because we've got all the services, we've got everything in place to do that. The provision of the real-time ultrasound, we've got specific allocation of time for those patients.*	*(Physiotherapist (Private Sector) 4)*
**4. Memory, attention and decision processes**	
*Every time the patient gets a request form for radical prostatectomy, … my secretary as a standing practice will actually refer to (a physiotherapist) in the private system. In the public system, they will go to a continence service. Or they also get given the option of going privately as well.*	*(Urological Cancer Surgeon 2)*
*He (the surgeon) said to go and see his receptionist, and she'll give you all the details. So she gave me (the physiotherapist’s) name, the phone number, and where… she said they're just around the corner so I rang up and made an appointment.*	*(Patient 4)*
*Well this (advice to undertake PFMT) was part of his (the surgeon’s) professional advice, that I (had) come to seek. So yes, I didn't think I had an option to say, 'No, I'm not going to do that,' it wasn't even part of my thinking.*	*(Patient 4)*
**5. Social Influences (Norms)**	
*… if you're going to put yourself in the hands of a medical professional, you're really a bit of a dill if you don't embrace, to all intents and purposes, everything that is being said and recommended to you.*	*(Patient 5)*
*… a few other colleagues who I'd spoke to had said (to) make sure you do your pelvic floor exercises, so I thought, gee this is really important.*	*(Patient 11)*
*I have two daughters who are science graduates from university, they took a great interest in it, and they were the first people to tell me about doing these exercises for the pelvic floor. And they said, 'You must practice before you go into hospital, Dad'. And I started doing it.*	*(Patient 12)*
**6. Beliefs about consequences**	
*The benefit (of PFMT) is it will reduce the impact of the surgery on their (patient's) symptoms, and the time course of their symptoms. But also, what you're also doing is it will reduce the impact of them not knowing what is likely to happen to them. And also, essentially what you're doing is reestablishing and improving the patient's locus of control.*	*(Physiotherapist (Private Sector) 5)*
*I was scared shitless (of PPUI), that's the long and the short of why I took it (PFMT) up, because I wanted to make it as easy as possible for myself.*	*(Patient 10)*
*It (cost) wasn't a consideration. I mean, I would have paid the earth provided I could get some guarantees that, you know, I'm going to come out as well as possible.*	*(Patient 10)*

As the domains ‘social/professional role and identity’, and ‘beliefs about capabilities’ proved important in guiding the direction of interviews, particularly for referrers and providers, these domains are presented first. Other theoretical domains are presented in descending order of relevance. Only those theoretical domains identified by participants as relevant to the study question and local clinical setting are presented.

### Social/professional role and identity and beliefs about capabiities

#### Referrers

Referrers’ perceptions of their professional role were significant barriers or enablers to preoperative PFMT. For example, *a priori*, it was hypothesised that GPs, as primary contact physicians, might strongly influence patients’ receipt of preoperative PFMT. Those GPs interviewed suggested, however, that their role in the management of prostate cancer related primarily to preliminary diagnosis and referral to specialist care. Subsequent referral to preoperative PFMT was the preserve of the urological cancer surgeon and their practice associates. GPs also felt that the path that patients followed to surgery circumscribed their role in preoperative work-up; once referred to the urologist for definitive diagnosis of localised prostate cancer, patients often did not re-present to the GP until after radical prostatectomy. Thus, without preempting the need for radical prostatectomy, GPs often *could not* refer patients for preoperative PFMT.

Urological cancer nurses were similarly bound by the practical limitations of their jobs, usually only seeing men postoperatively (e.g. for urinary catheter removal). While there was some capacity for private sector urological cancer nurses to see men preoperatively, this did not occur routinely but rather in an *ad-hoc* fashion in response to individual patients’ concerns. In the public sector, preoperative work-up for surgery was performed through a generic ‘preadmission clinic’, with no involvement of urological cancer nurses. Thus, while able to advise on PFMT when PPUI *presented* (after removal of the urinary catheter approximately two weeks after surgery), urological cancer nurses tended to be removed from preoperative work-up, including referral to PFMT. An exception was one private sector urological cancer nurse, who specifically nominated that it was her role to encourage receipt of preoperative PFMT, and accordingly arranged her work practices to see patients preoperatively.

All three urological cancer surgeons performed radical prostatectomies at both the public hospital and the co-located private hospital. The majority of patients were reviewed preoperatively by their surgeon within a private practice setting, regardless of whether surgery ultimately took place in the public or private hospital. As a rule, the surgeons implicitly assumed the role of determining appropriate work-up for surgery, including the need for preoperative PFMT. Indeed the surgeons professed to refer all patients, public and private, for preoperative PFMT – most commonly to a specific, local, private sector physiotherapy group. But the practicalities of making that referral (i.e. providing men with a letter of referral and/or the contact details of a provider of PFMT) were delegated to a practice receptionist or administrator. It was noted that a small number of public sector patients could not afford to consult a urological cancer surgeon except through a public urology clinic, and that these patients might be scheduled for surgery by a surgical registrar, without having been reviewed in person by a ‘consultant’ surgeon.

#### Providers

There were limited opportunities for providers to review patients preoperatively, and hence to provide preoperative PFMT, unless patients had received and acted upon a specific referral from their surgeon. For patients having radical prostatectomy in the public hospital, providers were absent from routine preoperative workup, including the preadmission clinic. Those patients having surgery in the private hospital did routinely consult a hospital-based physiotherapist (employed by the aforementioned local private sector physiotherapy group) within a preadmission clinic, albeit this was usually within the week of surgery and the consultation was focused on immediate postoperative care.

Particularly in the public sector, PFMT was seen as the preserve of staff employed in ‘continence’ roles (e.g. those working in specialised continence clinics or employed as, ‘women’s (sic) health and continence’ physiotherapists). Those working in public sector continence roles, though, did not routinely receive referrals to provide preoperative PFMT for male patients, and as such were unsure of, or did not perceive, a demand to do so. Nor were referrals sought; rather it was perceived that if preoperative PFMT were something that the surgeons desired, referrals, and/or direction from surgeons to set up a (male) PFMT service, would be forthcoming. Contrary to this, those private sector physiotherapists working in the outpatient setting identified a responsibility to both patient and urological cancer surgeon to provide preoperative PFMT for men having radical prostatectomy. Their ability to provide PFMT was still seen as contingent upon the patient having received a direct referral from the surgeon, this referral perceived as the key enabler.

#### Patients

Patients agreed that the role of referring to preoperative PFMT was the domain of the surgeon. Patients’ perceptions of who subsequently carried the responsibility for PFMT provision were informed by their surgeon’s advice (i.e. those patients specifically referred to a physiotherapist saw PFMT provision as a physiotherapist’s role). Those patients made aware of pelvic floor muscle exercises, yet who did not receive a surgeon’s referral to a provider, did not necessarily perceive a role for healthcare providers in PFMT provision. Rather, pelvic floor muscle exercises were seen as something that could be learned and practised independently.

### Knowledge/skills

The closely aligned theoretical domains ‘knowledge’ and ‘skills’, as they pertain to preoperative PFMT, encompass several discrete constructs: knowledge of the evidence for, and hence the effectiveness of, PFMT; (for the referrer) knowledge of to whom and how to refer for PFMT, (for the provider) knowledge/skills of how to provide PFMT, and (for the patient) knowledge of the benefits of PFMT and where and how to receive it.

#### Referrers

The three urological cancer surgeons all reported knowledge, albeit variable in depth, of the primary research evidence supporting preoperative PFMT for men undergoing radical prostatectomy. One even cited novel research conducted within his urology practice. Interestingly, the surgeons did perceive that unspecified surgical colleagues varied in their perceptions regarding the strength of the evidence (it was not clear whether they were referring to local colleagues), and the degree of benefit to patients in receiving formal PFMT. Urological cancer nurses reported limited knowledge of the primary research, but all espoused support for preoperative PFMT, primarily on the basis of their clinical experience.

In keeping with their perceived limited role in preoperative work-up, GPs’ knowledge of the evidence for/role of preoperative PFMT was limited to that garnered through the process of recruitment to the current study. It was even suggested by one GP that many of his GP colleagues had no knowledge that PPUI was a potential complication of radical prostatectomy, let alone of therapies to reduce the impact of said PPUI.

There was a marked discrepancy amongst referrers in their knowledge of private versus public sector providers of PFMT. Again, the urological cancer surgeons and their associated private sector urological cancer nurses reported having developed clinical ‘partnerships’ with a local private sector physiotherapy practice to provide PFMT. As regards local public sector providers of PFMT, however, knowledge was often limited and/or incorrect. No providers, for example, were aware of a recently established public university teaching facility that incorporated a PFMT service. Even when aware of local public sector providers (e.g. continence clinics), referrers professed reluctance to refer patients due to a lack of knowledge regarding the quality of the services provided. Noting that some patients presented from rural or regional areas, referrers were also unaware of providers outside of the local metropolitan area, in either of public or private sectors.

#### Providers

Unsurprisingly, potential providers’ knowledge of the evidence supporting preoperative PFMT was closely aligned to their provision of preoperative PFMT. Those private sector physiotherapists having partnered with the urological cancer surgeons to provide PFMT were able to cite specific randomised-controlled trials that informed their practice. Public sector physiotherapists and continence nurses on the other hand, with one exception, perceived a theoretical rationale for PFMT without explicit knowledge of published trials. As regards knowledge of how to provide preoperative PFMT, both physiotherapists and continence nurses noted that their undergraduate professional training did not encompass PFMT, and that external opportunities for postgraduate education and training in PFMT provision were limited. Several providers reported having attended a one-day course in male pelvic floor management, run by a private physiotherapy/nursing education group; otherwise education in the provision of PFMT was generally undertaken in the workplace, and only in response to workload demands.

#### Patients

Those patients not having received PFMT reported ignorance and/or a lack of appreciation of the potential severity of PPUI and the role of preoperative PFMT. On the other hand, being informed of PPUI and PFMT, particularly by the urological cancer surgeon, was a key enabler of provision/receipt. It was noted that there was limited public discussion/acknowledgement of urinary incontinence as a men’s health issue, hence, unless ‘educated’ by a healthcare provider, most patients were not to know of PPUI, let alone treatments thereof. The preoperative consultation with the urological cancer surgeon was often the only opportunity for patients to gain knowledge of PFMT before having radical prostatectomy, but the primary focus of this consultation was perceived to be cancer control rather than management of postoperative complications. Additionally, at least prior to the consultation with the urological cancer surgeon, urinary incontinence and PFMT were perceived as women’s issues, and not something men knew about or discussed in the public domain.

A consistent point raised by all participant groups, was that provision of information to patients about PPUI and the role of PFMT, even by the urological cancer surgeon, did not guarantee of transfer of knowledge. The timing of information, the method by which the information was given (e.g. verbally or in writing) and the relative emphasis placed upon the information, were all potential barriers or enablers to transfer of knowledge, and hence preoperative PFMT.

A minority of patients did acquire knowledge of PPUI and pelvic floor exercises from sources other than the urological cancer surgeon, including peers who had previously had radical prostatectomy, local prostate cancer support groups, prostate cancer-related web-sites, and family members. Thus-acquired knowledge, however, did not necessarily translate to consultation with a provider for preoperative PFMT. Indeed, without a referral from their surgeon to a *specific* provider, patients reported difficulty in finding a suitable physiotherapist or continence nurse.

### Environmental context and resources

The theoretical domain ‘environmental context and resources’ encompasses a broad range of practical constructs, such as the availability and management of staffing, physical space and equipment. Data relating to competing time and task constraints and the financial aspects of PFMT provision/receipt will also be presented under this domain. The three participant groups often concurred in their perceptions of environmental/resource barriers and enablers to PFMT - their results will thus be presented together.

Availability of staff was perceived as a key ‘environmental/resource’ determinant of the ability to provide PFMT. Providers in the public sector consistently reported that the pool of staff to provide healthcare services (e.g. physiotherapy, continence services) was constrained by department budgets. Expansion and/or development of PFMT services, e.g. to men having radical prostatectomy, would thus necessarily involve withdrawal of other services, or extension of waiting lists (the reduction of which was a key performance indicator). Contrary to this, the local private sector physiotherapists noted that, with establishment costs of staff training and equipment having been met, staffing could be adjusted to meet demand for preoperative PFMT. The private sector physiotherapists also had scope to extend their services outside of normal working hours (e.g. weekends, after-hours), whereas public sector providers could not.

Availability of physical space and equipment to provide preoperative PFMT was not a barrier in either of public or private sector settings. While individual providers used different equipment, e.g. real-time ultrasound, mirrors, tactile feedback, in their PFMT protocols, all had ready access to the equipment they deemed necessary to provide PFMT, and all had ready access to secluded treatment rooms.

Geographic proximity of patients to providers of PFMT, i.e. where patients lived, was also seen as a key determinant of preoperative PFMT provision/receipt. That the aforementioned local private sector physiotherapy group worked in a building adjacent to that of the three surgeons’ consulting rooms facilitated referral and provision – it was easy and timely for locally-based patients to walk across and book/attend appointments for PFMT. However, as the clinical setting in question was/is a tertiary referral centre, some patients had travelled from regional and rural areas for management of their prostate cancer. While the local private sector physiotherapists were often able to schedule regional/rural patients for PFMT immediately after the preoperative consultation with the surgeon, there was no scope for this to occur in the public sector – the continence clinics and university teaching facility were located in different suburbs, had waiting lists, and/or were open only to those living in specified metropolitan referral zones.

Referrers acknowledged that patients without private health insurance (approximately 25% of their patient load), both regional/rural and metropolitan, could access the local private sector providers of PFMT. It was perceived, however, that the financial cost of private sector PFMT (to patients) might be a significant barrier. Even for those patients with private health insurance, referrers suggested that the costs of radical prostatectomy and the attendant hospitalisation were substantial, and that ‘discretionary’ treatments such as preoperative PFMT, might thus be neglected. Interestingly, the private sector providers of preoperative PFMT, and those patients attending for preoperative PFMT, downplayed the issue of financial cost (discussed further in the section below, ‘Beliefs about consequences’).

The timing of the preoperative consultation(s) with the urological surgeon (in relation to the timing of surgery) was also posited to influence provision/receipt of, PFMT. Where patients had high-risk cancer requiring urgent surgery, one surgeon noted that there was a limited timeframe within which to consult a provider of PFMT. Several referrers and providers suggested that even those patients with a longer lead-time to surgery often felt under time–pressure in their work/life schedules, and thus might neglect recommendations for PFMT.

### Memory, attention and decision processes

The theoretical domain ‘memory, attention and decision processes’ normally relates to the factors that influence whether an individual healthcare provider would remember and choose to engage in an evidence-based healthcare practice, in this case to provide preoperative PFMT, when faced with an appropriate/eligible patient. None of the actual providers of preoperative PFMT interviewed in this study noted any barriers to preoperative PFMT, *once the patient had scheduled a consultation.* As such, this section of the results will focus on those factors influencing referrers’ and patients’ memory, attention and decision processes (to refer patients for PFMT and to act on such a referral, respectively).

The three urological cancer surgeons had all instituted systems within their private practice settings to ‘automate’ and direct referral of patients for preoperative PFMT. As previously noted, these systems relied on reception/administrative staff to provide patients with the contact details of a PFMT provider, together with their hospital booking forms (for surgery), immediately after the decision to have radical prostatectomy had been made. None of the surgeons knew exactly how these contact details were provided (e.g. on a business card, on a referral form), and no such system of referral existed in the public urology clinic. It was noted, too, that when busy, the reception staff might forget to provide the contact details.

Patients were more likely to act on the referral if they remembered the surgeon having discussed and emphasised the importance of preoperative PFMT when the decision for radical prostatectomy was made. The emotional impact of having been diagnosed with cancer, however, made it difficult for patients to take in information on PFMT. And facing a cancer diagnosis, patients tended to minimise the importance of potential side effects of surgery, like PPUI, rather planning to confront these postoperatively if/when they occurred.

Patients also varied considerably in their preferences for information (volume and mode of delivery) about prostate cancer and related issues, including PFMT. Some preferred written information that could be reviewed at home, others felt that ‘too much information’ was presented in this way, and thus was likely to be ignored. The presence of a family member or partner at the preoperative consultation with the urological cancer surgeon was seen as an enabler of preoperative PFMT, insofar as they could help patients remember the surgeon’s advice/recommendations, and were often more focused than patients on postoperative quality-of-life.

### Social influences (norms)

The theoretical domain ‘social influences (norms)’ refers to the extent to which social influences, including peers, managers, other professional groups, patients and relatives, facilitate or hinder (in this instance) preoperative PFMT.

#### Referrers

While the three urological cancer surgeons worked within the same public hospital Department of Urology and in neighbouring private consultation suites, there is no evidence that this influenced their practices of referring patients for PFMT. Indeed, the surgeons claimed to be unaware of their colleagues’ referral practices. The stated decision to refer patients for preoperative PFMT (or otherwise) was that of the individual surgeon, and based on their views of the research and clinical evidence.

#### Providers

As noted, the urological cancer surgeon was the main ‘social’ influence on providers’ ability to provide PFMT, by virtue of their role as primary referrer. The influences of peers and management, however, also acted as both barriers and enablers to providers’ provision of PFMT. Given the aforementioned staff constraints within the public healthcare settings, development of new (or extension of existing) clinical services was seen as difficult, and without push from management, unlikely to occur. In the public hospital physiotherapy department particularly, PFMT provision had traditionally been the domain of female therapists, for female clients. As there had been no pressure from urologists on management to institute a PFMT service for male patients, this was not a management or clinician priority. In the private sector physiotherapy group, however, and to a lesser extent the public sector continence clinics, provision of PFMT to patients having radical prostatectomy was seen as part of the organisational charter, and peer-training occurred in order to sustain a core group of providers.

#### Patients

Patients consistently reported that the advice, recommendation or mandate of the urological cancer surgeon was the primary influence on their decision to undertake preoperative PFMT. Peers and family members, however, also influenced their decision. It was common for patients to report a peer-group of men with prostate disease, including cancer; the experiences and opinions of these men, and their recommendations for or against PFMT, were highly valued. Two patients noted that the impetus for them to undertake preoperative PFMT was advice received through local prostate cancer support groups. Participants from all groups suggested that patients’ wives/partners and daughters often directed their husbands/fathers to attend PFMT.

### Beliefs about consequences

#### Referrers and providers

Referrers and providers consistently discussed the consequences of referring for/providing preoperative PFMT in terms of the benefits accruing to individual patients (e.g. a reduction in severity and duration of incontinence, consequent improvements in quality of life). Additional enablers to referral/provision of PFMT were referrers’/providers’ perceptions of the psychological benefits (to patients) of having a ‘team approach’ to cancer care, of patients being adequately mentally prepared for PPUI, and knowing that they (the patients) had done all they could to prepare for radical prostatectomy. Notwithstanding the aforementioned potential issues of financial costs/time burden to patients, providers/referrers reported no adverse consequences to patients of receiving preoperative PFMT.

The sole negative consequence of providing preoperative PFMT, as identified by public sector providers, was the burden that this may place on already ‘stretched’ staff resources, and consequent impacts on other services, e.g. an increase in waiting lists, an inability to meet key performance indicators for patient waiting times. Both public and private sector providers noted the professional satisfaction gained from providing evidence-based healthcare practices; another positive consequence for providers of preoperative PFMT, nominated by those in the private sector, included the inherent satisfaction gained from helping this particular patient population, i.e. men of a certain age, and at a vulnerable time (following diagnosis of prostate cancer). One physiotherapist noted that provision of PFMT was also less physically demanding than other components of their job, and made for a welcome variation in the workday.

#### Patients

Save for potential financial cost, time cost and travel burdens, patients similarly expressed no negative consequences of receiving preoperative PFMT. Rather, the potential costs of not receiving PFMT (i.e. PPUI and associated embarrassment, physical discomfort, social implications) were emphasised by patients as enablers of PFMT. While the emotional burden of a cancer diagnosis has been noted as a potential barrier to patients taking in information on PFMT, several patients reported the cancer diagnosis as being a ‘spur’ to preoperative PFMT; by engaging in PFMT they were doing everything they possibly could to ensure a positive outcome. Concerns about financial costs were often minimised in this context.

While not described by patients themselves, several providers/referrers suggested that a barrier to preoperative PFMT was that patients might assume a low-risk for PPUI (i.e. perceive that they might be ‘lucky’). Such patients might thus only seek to receive PFMT if/when PPUI presented postoperatively, perceiving no negative consequence in delaying PFMT.

### Additional patient-related barriers

Several additional ‘patient-related’ barriers to PFMT were raised by referrer and provider participant groups, which do not fit comfortably within the listed theoretical domains. These barriers related primarily to perceived characteristics of individuals or groups of patients, precluding uptake/receipt of PFMT even in the circumstances of having received a referral for the same, to a cost-free provider within geographical proximity.

Some patients were perceived as being disinterested in the recommendations of their urological cancer surgeons. Some patients were perceived as experiencing embarrassment or shame that would preclude their seeking care/treatment for PPUI, and/or care involving the pelvic floor/penile region. Contrary to this perception, however, the majority of patients interviewed suggested that having undergone investigation for prostate cancer, typically involving digital rectal examination and transrectal biopsy, embarrassment was not (or no longer) an issue. Non-English speaking, or ‘culturally diverse’ patients were also seen as less likely to present for PFMT.

## Discussion

The primary aim of the current study was to investigate barriers and enablers to preoperative PFMT amongst men having radical prostatectomy, to inform the development of a multifaceted behaviour change intervention to improve PFMT provision/receipt. As such, this discussion will focus on those barriers and enablers perceived to be most significant in determining provision/receipt of preoperative PFMT within a local clinical setting, and those barriers and enablers perceived to be readily amenable to intervention. Potential strategies to overcome identified barriers will also be described.

Ostensibly, the majority of patients having radical prostatectomy at either of the tertiary referral public hospital and the geographically co-located private hospital, *could* access a provider of preoperative PFMT. All patients living within the local metropolitan area had both public (i.e. no financial cost) and private sector provider ‘options’; those living in regional/rural areas had access to a private sector physiotherapy provider. Furthermore, according to the urological cancer surgeons, approximately 75% of patients having radical prostatectomy within the study setting had their operation in the private hospital, and 100% of these ‘private’ patients were referred for preoperative PFMT.

Notwithstanding that small group of public-sector patients living in regional/rural areas, and for whom the cost of private PFMT might be prohibitive, there was/is an apparent mismatch between urological cancer surgeons’ stated referral patterns (which align with published recommendations) [17] and patient receipt of preoperative PFMT. None of the interviewed ‘providers’, including all three *actual* public sector providers of PFMT to men, reported turning patients away. As such, it would seem that the gap between published recommendations and observed practice relates predominantly to slippage between the surgeon’s ‘referral’ of a patient for PFMT, and the subsequent receipt of that referral by a provider, to then schedule a consultation. It is well-established that engaging men in preventive healthcare is difficult, for manifold reasons [22], albeit it should be noted that the current study explicitly concerns men who have already ‘opted in’ for preventive care, at least insofar as they are receiving active treatment for prostate cancer.

A strong recommendation or mandate from the urological cancer surgeon was seen as essential to patients’ ultimate receipt of preoperative PFMT. Excepting within the hospital preadmission clinic, the urological cancer surgeon (or the surgical registrar) was the only healthcare professional *routinely* seen by all patients between definitive diagnosis of localised prostate cancer and having radical prostatectomy. The consultation with the urological cancer surgeon thus represents the only *consistent* opportunity for men to be informed, in a timely fashion, of: (i) the risk of PPUI; (ii) the role of preoperative PFMT; and (iii) who can provide preoperative PFMT. This consultation with the urological cancer surgeon, however, is often focused on ‘cancer control’ – the relative mortality-benefits of e.g. radical prostatectomy versus active surveillance versus radiotherapy. The potential complications of treatment, e.g. PPUI, and the role of PFMT, might thus be afforded less import, by the patient if not the surgeon. Previous research has shown that treatment seeking in men with urinary incontinence is linked to symptom severity [23], and PPUI, by definition, only occurs postoperatively.

Notwithstanding that the urological cancer surgeons describe advising/informing all patients of the likelihood of PPUI, and the benefits of preoperative PFMT, some patients denied having received this information. There is an apparent disconnect between the information patients receive and retain in the context of the preoperative consultation. It is also noteworthy that the surgeons had no established method of discerning, before the surgery, whether patients had actually received preoperative PFMT. Without some reinforcement, e.g. by a urological cancer nurse, or with written information, it is possible (likely) that patients may forget or neglect their surgeon’s advice. Provision of a provider’s contact details, together with surgical booking forms and sundry administrative paperwork, e.g. by a practice receptionist, would appear a suboptimal mode of ‘referral’.

Novel strategies may be required to improve: (i) patients’ retention of information provided by the urological cancer surgeon; and hence (ii) patients’ uptake of surgeons’ referrals for preoperative PFMT. Several patients reported being disoriented when advised of their prostate cancer diagnosis, however those who had an opportunity to reflect on the diagnosis before deciding to have radical prostatectomy (at a subsequent consultation) were more likely to receive preoperative PFMT. In lieu of potentially costly repeat consultations with the surgeon, some form of follow-up/reinforcement of education, e.g. via telephone with a urological cancer nurse, or simple written educational materials that could be read at home, might reduce patients’ knowledge barriers to PFMT.

We have described public sector, cost-free service options for preoperative PFMT in our clinical setting (continence clinics, the university teaching facility). It was difficult for patients to access these services however, as the surgeons were either unaware of the services, or, with one exception, had not developed a clinical relationship with the services, and hence did not refer to them. Nor did the public sector services actively publicise to referrers or patients their ability to provide preoperative PFMT to men having radical prostatectomy. Public sector providers may lack incentives to develop such clinical relationships with urological cancer surgeons – those interviewed had a full workload of other ‘continence clients’ - and indeed potential increases in workload (should more patients be referred), with no promise of commensurate increases in staffing, might actively work as a disincentive.

Financial cost and lack of private health insurance are established barriers to PFMT amongst women with urinary incontinence [24]. No patient interviewed for the current study explicitly declined preoperative PFMT because of financial cost, rather ignorance was reported as the primary reason for non-receipt – that they were not informed of the benefits of PFMT. Our preliminary audit did find lower receipt of preoperative PFMT among public sector patients, which one might expect to be related to cost factors. It is possible, too, that urological cancer surgeons may place less emphasis on preoperative PFMT for public patients, perceiving there to be no, or limited, provider options for these patients.

Additional strategies may be required to improve provision/receipt of preoperative PFMT amongst public sector patients. While there is limited scope in the studied clinical setting to alter public sector provider funding and/or staffing priorities, surgeons’/referrers’ knowledge of the *existence* of public sector providers might be addressed, e.g. by means of a provider ‘directory’, which includes the contact details and method of referral to those providers. It may be incumbent on external persons/professional organisations to develop such a directory, and/or to act as an intermediary between referrers and public sector providers (those in the continence clinics and the university teaching facility), to aid establishment of clinical relationships. Alternatively, new clinical relationships might be developed between the urological cancer surgeons and heretofore only ‘potential’ providers of PFMT (e.g. those physiotherapists working in the outpatient setting at the public hospital). Benefits of this latter approach include the geographical location – public patients could walk to access preoperative PFMT. Education and training in PFMT provision would, however, be required.

One proposed method of improving and coordinating patients’ access to prostate cancer services, e.g. of preoperative PFMT, is the use of specialist ‘prostate cancer nurses’ [25]. The Prostate Cancer Foundation of Australia, a registered charitable trust, is currently undertaking a three-year trial of such prostate cancer nurses, including in the current clinical setting. At the time of study conduct, however, no local prostate cancer nurse had been appointed.

### Strengths and limitations of the study

While previous studies have investigated barriers to receipt of and adherence to PFMT in women with urinary incontinence [24,26], the current study is the first to investigate barriers and enablers to PFMT specifically for men, a noted research gap [27]. Additionally, whereas previous studies have focused exclusively on patient-related and described barriers to PFMT, the current study also investigated barriers from referrer and provider perspectives. All key local referrers and providers were invited to participate in the study, and we would argue that we had achieved data saturation with these participant groups. In retrospect, additional interviews with the reception/administrative staff of the urological cancer surgeons and the public hospital urological clinics might have provided valuable data on surgeons’/registrars’ referral processes.

One limitation of the study is that of the 13 patients interviewed, ten had radical prostatectomy with the private hospital setting, and only three in the public hospital setting. This is reflective of actual public:private patient ratios at our centre, however a greater number of ‘public’ patient participants may have provided more complete data on their specific barriers/enablers to PFMT. We note that all public hospital patients responding to the study invitation were interviewed, but also that non-English speaking patients were excluded from participation, likely a greater proportion of public than private patients. Also of note, the majority of private patients interviewed for the study had received preoperative PFMT, perhaps representing a participation bias. It is likely that the barriers to preoperative PFMT coincide with barriers to study participation (e.g. lower literacy and/or socio-economic status, and a reticence to discuss matters urological).

The current study investigated the barriers/enablers to preoperative PFMT in one specific clinical setting (i.e. a tertiary referral setting in Sydney, Australia). Different barriers may exist in other clinical settings/geographical locations. As argued by McCluskey and Middleton, however, ‘local’ barriers must be identified when seeking to develop tailored interventions to address evidence-practice gaps, in this case suboptimal provision/receipt of PFMT [28]. Some of the barriers/enablers raised by participants are likely to be common across healthcare settings where allied health services are recommended before surgery, or where access to ‘specialised’ allied health services is dependent on medical referral.

A consideration when interpreting the results of the current study was the primary researcher’s (ADH’s) concurrent work role as a physiotherapy manager/clinician, and the potential influence of this on participants’ responses. All participants were advised before interviews that no judgements would be made regarding their responses and/or knowledge regarding preoperative PFMT. A further limitation of the study is that, following the generation of preliminary themes from transcribed interviews, ADH was solely responsible for all indexing of participants’ responses into theoretical domains, charting, and mapping/interpretation of interview data. This process, however, was overseen by one of the other researchers (GSK). Cross-checking of the indexing and charting processes, and involvement of a ‘referrer’ or ‘patient’ in the mapping and interpretation process, may have provided additional and/or different insights. Arguably though, as the study was conducted to inform a physiotherapist-led intervention to improve provision/receipt of preoperative PFMT, a physiotherapist-led analysis was appropriate.

## Conclusions

The results of this study contribute to a better understanding of why men having radical prostatectomy may not receive preoperative PFMT, with implications for the planning of a behaviour change intervention to improve provision/receipt of preoperative PFMT in the local clinical setting. Such an intervention would need to address: (i) how urological cancer surgeons convey information to patients on the role and importance of preoperative PFMT; and (ii) how patients obtain information about and make contact with providers of preoperative PFMT, particularly in the public sector. Other barriers to preoperative PFMT may exist in other clinical settings, e.g. rural/regional settings, where there may be a dearth of PFMT providers, or where public sector patients predominate, warranting further research.

## Abbreviations

GP: General practitioner; PFMT: Pelvic floor muscle training; PPUI: Post-prostatectomy urinary incontinence.

## Competing interests

ADH is employed as a physiotherapist within Mungovan Breckenridge Physiotherapy & Associates, a private physiotherapy services group in Western Sydney, Australia.

GSK and AJB have no competing interests to report.

## Authors’ contributions

ADH, GSK and AJB participated in the study conception, design and coordination. ADH conducted the interviews and data analysis. ADH wrote the first draft of the paper with input of all authors. All authors read and approved the final manuscript.

## Pre-publication history

The pre-publication history for this paper can be accessed here:

http://www.biomedcentral.com/1472-6963/13/305/prepub
